# Ring enhancing lesions in the brain of an HIV infected patient: a diagnostic challenge

**DOI:** 10.11604/pamj.2017.26.185.9127

**Published:** 2017-03-30

**Authors:** Joe James, Nallaveettil Kesavan Thulaseedharan

**Affiliations:** 1 Department of Internal Medicine, Government Medical College Kozhikode

**Keywords:** Cerebral toxoplasmosis, magnetic resonance imaging, HIV, basal ganglia, mild cognitive impairment, gait apraxia

## Image in medicine

A 58-year old male presented with headache, unsteadiness of gait and cognitive decline in the form of loss of recent memory of 1-month duration. He also reported significant loss of weight. Neurological examination revealed short term memory loss and gait apraxia. There were no cerebellar signs or sensorimotor deficits. He was tested positive for HIV. MRI showed T2-FLAIR hyperintense lesions with edema in the left centrum semiovale (A), right basal ganglia (B) and right frontal corticomedullary junction (C). The corresponding lesions in T1 after gadolinium showed ring enhancement (D-F). His CD4 count was 70/µL. Serum IgG Toxoplasma was positive. He was started on pyrimethamine with sulfadiazine and anti-retroviral treatment and discharged. Toxoplasmosis is a parasitic infection caused by Toxoplasma gondii. CNS Toxoplasmosis is an important differential diagnosis in HIV patients presenting with neurological symptoms. Common presentations are headache, seizures, focal neurological deficits like hemiparesis or aphasia, and altered sensorium. Toxoplasmosis in AIDS is due to reactivation of previously acquired infection as immunity wanes, almost always when CD4 count is less than 100/µL. Multiple ring enhancing lesions in the corticomedullary junctions of frontal and parietal lobe, centrum semiovale and basal ganglia with edema and mass effect in an HIV patient is virtually diagnostic of toxoplasmosis. Ring enhancing lesions in the brain can also be seen in primary CNS lymphoma which often causes diagnostic confusion. An isolated ring enhancing lesion larger than 4cm in size or a positive serology for Ebstein-Barr virus in CSF favors lymphoma over toxoplasmosis.

**Figure 1:**
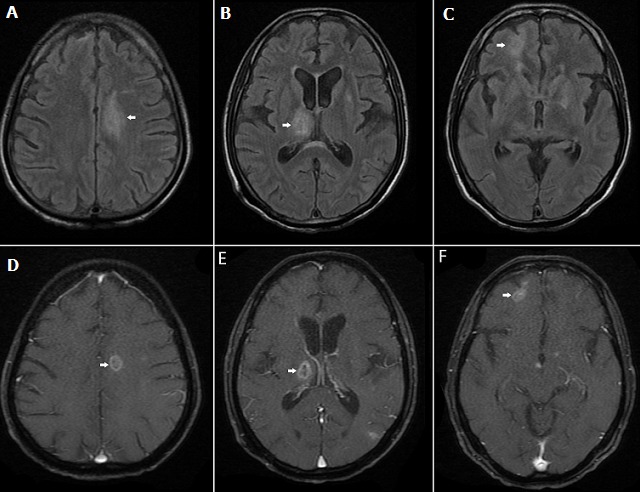
MRI Brain in CNS Toxoplasmosis

